# Quality Evaluation of Locally Manufactured Paracetamol Tablets in East Africa

**DOI:** 10.1155/2024/9437835

**Published:** 2024-09-14

**Authors:** Gerald Marisa, James Kapala, Tanga Mafuru, Raphael Matinde, Emmanuel Kimaro, Eliangiringa Kaale

**Affiliations:** ^1^ Department of Pharmaceutics Catholic University of Health and Allied Sciences (CUHAS), Mwanza, Tanzania; ^2^ Department of Medicinal Chemistry Catholic University of Health and Allied Sciences (CUHAS), Mwanza, Tanzania; ^3^ Pharm R&D Laboratory School of Pharmacy Muhimbili University of Health and Allied Sciences (MUHAS), Dar es Salaam, Tanzania; ^4^ Department of Medicinal Chemistry Muhimbili University of Health and Allied Sciences (MUHAS), Dar es Salaam, Tanzania

## Abstract

**Background:** Paracetamol, also known as acetaminophen, is categorized as an analgesic and antipyretic medication and is available as over the counter (OTC) medication. It is commonly used in conditions associated with pain and fever. There is a tendency for community to prefer using imported paracetamol tablets from Europe and United States than from Asia and Africa worrying of the quality of the products. Safety, effectiveness, and efficacy of a medicine can be guaranteed when its quality is reliable; however, there is limited data on the quality of locally manufactured paracetamol tablets, thus necessitating this study.

**Aim:** This study is aimed at assessing the quality of paracetamol tablets 500 mg manufactured by local companies by evaluating their physical parameters, assay results, and dissolution profiles. The compliance of these tablets with the specifications outlined in the British Pharmacopoeia (BP) was analyzed. Additionally, a comparative dissolution test was conducted to assess dissolution profile for innovator product and generics.

**Method:** Five different brands from East African countries with 76 tablets from each brand were compared with the innovator product regarding weight variation, hardness, friability, assay, and dissolution test based on the BP specifications.

**Results and discussion:** All samples of paracetamol tablets 500 mg from the local manufacturers in this study met the specifications set by the BP for physical parameters, including weight variation, friability, hardness, and disintegration tests. The weight variation test, directly related to drug content variation, demonstrated compliance within the acceptable deviation of 5%. Similarly, the assay test, which determines the concentration of the active pharmaceutical ingredient (API), confirmed that all samples complied with the acceptable concentration range of 90%–110% for paracetamol. The dissolution test, assessing the percentage release of the API within a specified time, demonstrated that at 15 min, two samples (diodol and enamol) exhibited lower concentration releases than the required 80%, indicating potential delays in their bioavailability and onset of action.

**Conclusion:** To conclude, all samples had good quality and they can be used for their therapeutic purposes.

## 1. Introduction

Paracetamol, also known as acetaminophen, is categorized as an analgesic and antipyretic medication [1]. It is used worldwide and is available without a prescription, making it a common household drug. It has various applications, including reducing fever, relieving headaches and minor aches, and being a key ingredient in cold and flu remedies [2]. In combination with opioids, it is used for severe pain management [3]. While generally safe at recommended doses, overdose can cause potentially fatal liver damage [4–7]. The overdose is mainly managed by using activated charcoal and N-acetylcysteine; rarely, paracetamol toxicity leads into death [8, 9].

The chemical name for paracetamol is 4′-hydroxyacetanilide or N-(4-hydroxyphenyl)acetamide. It has a molecular formula of C_8_H_9_NO_2_, a relative molecular mass of 151.2 g/mol, and appears as a white, odorless crystalline powder which has a melting point of 169 °C [1]. It is sparingly soluble in water but freely soluble in ethanol TS (~750 g/l) and acetone R while being practically insoluble in ether [10]. Proper storage of paracetamol requires a tightly closed container and protection from light.

Different drug formulations may lead into difference in terms of drug performance, safety, and efficacy. Ensuring the reliability and quality of pharmaceutical dosage forms, including tablets containing paracetamol, is crucial.

The previous studies conducted in Addis Ababa and the Somali region of Ethiopia show that there is a presence of paracetamol that did not comply with the pharmacopeia requirements [11, 12]. Evaluation of quality attributes for different paracetamol generics is one of the ways of ensuring paracetamol used by community is of good quality and will provide accepted therapeutic outcomes [4, 11].

The quality of a product refers to its ability to meet given requirements, and reliable standards are essential for producing acceptable products [13]. To evaluate the quality of pharmaceutical dosage forms, evaluation tests specified in official monographs such as the United States Pharmacopeia (USP) and BP must be performed [13]. Tests such as weight variation, content uniformity, thickness, hardness, friability, disintegration, dissolution, and assay are crucial in assessing tablet quality [13, 14].

The British Pharmacopoeia outlines several parameters for quality testing of tablets. Hardness refers to the crushing strength of a tablet, assessing its ability to withstand force without breaking. Friability measures the percentage of weight loss due to mechanical action, indicating a tablet's resistance to fracture during transport. Disintegration refers to the complete breakdown of a tablet into fragments or soft mass in a specific time frame and is a guide to comparative bioavailability. The dissolution test determines the drug release pattern over time, while assay measures the content of the active pharmaceutical ingredient (API) in a product [15–17]. There is a tendency for community to prefer using imported paracetamol tablets from Europe and United States than from Asia and Africa worrying of the quality of the products. Safety, effectiveness, and efficacy of a medicine can be guaranteed when its quality is reliable; however, there is limited data on the quality of locally manufactured paracetamol tablets, thus necessitating this study.

## 2. Materials and Methods

### 2.1. Materials

Six samples of different paracetamol brands were collected from community pharmacies in Mwanza City, Tanzania, and were prepared for the general and specific tests.

Reagents which were used include water (Sigma-Aldrich, Milli-Q water purification system, United States), methanol (Sigma-Aldrich, HPLC grade, United States), 0.1 N sodium hydroxide, (Sigma-Aldrich, HPLC grade, United States), and phosphate buffer pH 5.8.

Apparatuses used include Pyrex measuring cylinder (Pyrex, United States), class A volumetric flask (Duran, Germany), and Pyrex beaker (Pyrex, United States). Friability was determined using Copley friability tester and weight variation test was done using Ohaus analytical balance while hardness, thickness, and diameter were measured using Copley hardness tester.

Sonication was achieved using a sonicator (Branson SFX150 Sonifier, Branson, United States). UV-VIS spectrophotometer (Bioevopeak) was used for quantitative determination of API of the paracetamol samples collected. pH meter (Mettler Toledo Seven Compact S220 pH Meter, Mettler Toledo, Switzerland) was used for measuring pH of a buffer. Dissolution was performed using DT 800 dissolution apparatus 1 (Erwekar) (basket) to provide critical in vitro drug release information of both innovator product and generic products and to predict in vivo drug release profiles.

### 2.2. Methods

#### 2.2.1. General Tests

##### 2.2.1.1. General Appearance

Twenty paracetamol tablets 500 mg of each sample were unpacked and physically inspected to determine their color, shape, texture, and size.

##### 2.2.1.2. Weight Variation

Weight variation is used to show the uniformity of content of the tablet. Twenty tablets 500 mg of each sample were weighed using analytical balance, and the average weight and standard deviation were calculated.

According to USP, for tablet weighing greater than 325 mg, there should not be more than two tablets deviating from the average by more than 5%. This procedure was repeated in a triplicate.

##### 2.2.1.3. Friability Test

As from the BP, the test is used to determine how well tablets can stand up to coating, packaging, shipping, and other harsh conditions. Twenty tablets 500 mg of each sample were weighed on the analytical balance and then placed in the friability tester which rotated at 100 rpm. Finally, the tablets were dusted and reweighed again. The difference in weight was determined, and percent loss in weight was calculated. The procedure was repeated three times, and the average was computed.

##### 2.2.1.4. Hardness Test

Five tablets 500 mg of each sample were subjected for hardness tester, and the crushing strength of the tablet was measured. The average hardness of the tablets was calculated, and standard deviation was determined. This procedure was then performed in triplicate.

##### 2.2.1.5. Disintegration Test

As from the BP, the test has been used to determine the time required for the tablet to disintegrate. Six tablets 500 mg of each sample were placed in disintegration apparatus, where the volume of disintegration medium was 900 mL of water maintained at 37 ± 1°C as suggested by BP monograph. The time taken to break each tablet into small particles and pass through the mesh was recorded, and finally, the average time was calculated. The procedure was repeated thrice.

#### 2.2.2. Specific Test

##### 2.2.2.1. Dissolution Test

In compliance with the requirements for monographs of the BP in the dissolution test for tablets and capsules, 2.900 mL of phosphate buffer (pH 5.8) was used as a medium. From the dissolution tester, the paddle was set at 50 rpm. A sample of 20 mL of the medium was withdrawn and filtered. Filtrate was diluted with 0.1 M sodium hydroxide to make about 0.00075% *w*/*v*. Absorbance of this solution was measured at 257 nm using 0.1 M sodium hydroxide in the reference cell. The total content of the paracetamol was calculated, in the medium taking 715 as the value of A (1%, 1 cm) at the maximum at 257 nm. A triplicate measurement was done.

##### 2.2.2.2. Assay

Twenty paracetamol tablets 500 mg of each sample were weighed and pulverized by mortar and pestle. A quantity of the powder containing 0.15 g of paracetamol was added to 50 mL of 0.1 M sodium hydroxide, diluted with 100 mL of water, and shaken for 15 min, and sufficient water was added to produce 200 mL. The mixture was well mixed and filtered. Then, 10 mL of the filtrate was diluted with water to 100 mL. Ten milliliters of the resulting solution was added to 10 mL of 0.1 M sodium hydroxide and diluted to 100 mL with water, and finally, the absorbance of the resulting solution was measured at the maximum at 257 nm. The content of the paracetamol was calculated, taking 715 as the value of A (1%, 1 cm) at the maximum at 257 nm. This procedure was conducted in triplicate.

## 3. Results and Discussion

### 3.1. General Results

All tablets which were physically inspected were undamaged and smooth and in a uniform color and had similar features to reference Panadol from GlaxoSmithKline (GSK).

The results of physical parameters on all samples of paracetamol tablets 500 mg from the local manufacturers complied with the specifications in the BP and with an innovator product from GSK (Panadol).

### 3.2. Specific Results

#### 3.2.1. Physical Parameters

##### 3.2.1.1. Weight Variation

All the samples in the study passed the test as they complied with the BP specifications (Table 1). The essence of checking the weight variation of an individual tablet is directly corresponding to the variation in the drug content. The acceptable deviation should not be more than 5%; this is according to the BP specifications.

##### 3.2.1.2. Friability Test

All samples in the study passed the test as they complied with the BP specifications as shown in Table 1. It indicates the resistance of the tablets to external pressure from manufacturing, shipping, and transportation. From the BP specifications, there should be no more than 1% weight loss.

##### 3.2.1.3. Hardness Test

It is a measure of the compression force required to break a tablet. From the BP specifications, there should be no less than 50 N value of hardness. The study showed that the average compression force on each sample is above 50 N as shown in Table 1 and thus complied with the required specifications.

##### 3.2.1.4. Disintegration Test

It is the time taken for the completion of process of breaking down a tablet into smaller particles. The BP specifications require that for uncoated tablets, it should disintegrate in less than 15 min. All samples passed the test as they complied with the BP specifications as shown in Table 1.

##### 3.2.1.5. Assay Test

It is used to determine the concentration of the API in a sample. From the BP, the accepted concentration of paracetamol in a sample should be ranging from 90% to 110%. Assay results have shown that all tested samples of paracetamol tablets 500 mg from the local manufacturers complied with the specifications of the British Pharmacopoeia as with Panadol (refer to Table 2).

However, on statistical analysis, it was observed that there was a significant difference between the cetamol brand and the reference Panadol (*p* = 0.02) as shown in Table 3.

##### 3.2.1.6. Dissolution Test

**Figure 1:**
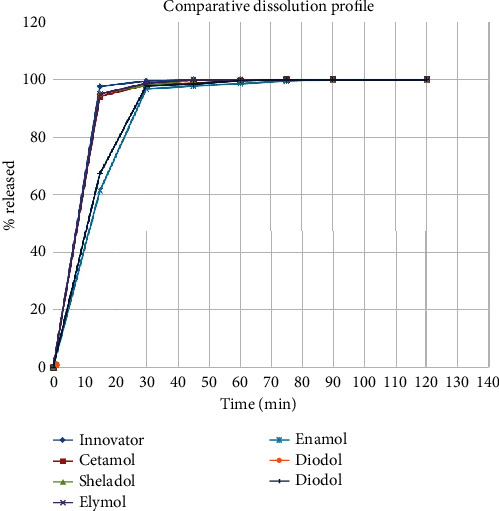
Comparative dissolution profile between paracetamol generics and innovator product.

The dissolution results indicate that at time 15 and 30 min, two samples deviated from the innovator values but from 45 to 120 min, there are superimposition of each sample and the innovator values as shown in Figure 1 and in Table 4. The dissolution at 30 min indicated a significant difference between the enamol brand and the reference Panadol (*p* = 0.03) as shown in Table 5.

### 3.3. Discussion

Pharmaceutical quality evaluation for the locally manufactured drug is important to be performed thoroughly to make sure each paracetamol tablet used by the community will provide the same efficacy as expected. A total of five different generics together with the innovator product (Panadol from GSK) were tested as per the BP specification recommendations.

The weight variation test is one of the tests used to control the manufacturing process and content uniformity because any variation in weight may significantly affect the amount of active ingredients in the individual tablets [12]. Results for the weight variation test have revealed that all tested samples have passed the test as per the BP specification recommendations.

All five generics and innovator product could resist abrasion when subjected to vigorous mechanical stresses from collision and tablet sliding towards one another and other solid substances which can result in the removal of small fragments from the tablets' surface. The results on friability and hardness tests for all generics and innovator product met the BP specifications as shown in Table 1.

The disintegration test is useful for assessing the potential importance of formulation and process variables on the biopharmaceutical properties of the tablets and as a control procedure to evaluate the quality reproducibility of the tablet during production [12]. The results on disintegration show that all tested samples comply with the test because the time to reach the endpoint was below a given limit as per the BP specification recommendations.

The difference factor (f1) is used to quantify the relative error between two curves at each time point. It calculates the percent difference between the curves, and an acceptable range of 0 to 15 indicates similarity. Values of 16 and above suggest dissimilar properties between the curves [18].

Additionally, the similarity factor (f2) is employed to measure the similarity in percent dissolution between the curves. It is derived from a logarithmic reciprocal square root transformation of the sum of squared errors. For the curves to exhibit similar characteristics, the acceptable range for f2 is 50 to 100. Values below 49 indicate dissimilar properties between the curves [19].

According to the data presented in Table 6, the comparative dissolution profiles of paracetamol tablets 500 mg exhibit identical characteristics. This indicates that all the curves in the graph are comparable and the products are suitable for therapeutic purposes. Consequently, these tablets can be used interchangeably, ensuring consistent effectiveness.

The dissolution test is one of the tests directly related to the bioavailability and performance of the product under in vivo conditions [12]. Two out of five generic products showing low amount were released in 15 min, but the released amount increased in 30 min to meet the recommended amount as explained by the BP. The results indicate that there is no significant difference between the enamol brand and the innovator product. This result is in congruence with the results from Gondar City, Northwest Ethiopia [11].

The results of an assay for the paracetamol active ingredients (Table 2) in paracetamol tablets show that there were no tested samples that failed to comply the BP specifications; this means that all manufacturing process was carried out correctly, and this will ensure the same efficacy among different generics tested and innovator product. However, the statistical test indicates a significant difference between the cetamol brand and the innovator product.

### 3.4. Study Limitation

This was a cross-sectional study which included the samples of products that were on the market during the time of the study. Therefore, it may not be a good reflection of the general trend of all time products available for community consumption. It thus may be necessary to conduct a follow-up study over the extended period.

## 4. Conclusion

All samples complied with the BP specifications on physical parameters, assay, and dissolution. This means all sample drugs are of good quality and can be used interchangeably for their treatment purposes. But since in this study there was a comparative dissolution profile between the locally manufactured paracetamol and Panadol (innovator), two samples (diodol and enamol) failed to release the required percent concentration of API; therefore, they failed the test.

## 5. Recommendations

Tanzania Medicine and Medical Devices Authority (TMDA) should ensure regular checking of the quality of the locally manufactured paracetamol tablets 500 mg in the market. Manufacturers should be informed on ensuring consistent production of good quality medicines by tightening up ranges of values required by either the BP or USP specifications.

## Figures and Tables

**Table 1:** Results for the tested physical parameters and specifications.

**Specifications**	**Physical parameters**	**Samples**						**Remarks**
		**Diodol**	**Enamol**	**Sheladol**	**Cetamol**	**Elymol**	**Panadol (ref)**	
White	Appearance	White	White	White	White	White	White	Pass
<5%	Weight Variation	4.7	4.7	4.6	4.3	1.3	1.9	Pass
<1%	Friability	0.35	0.49	0.5	0.26	0.11	0.05	Pass
<15 min	Disintegration	1.0	9.0	0.2	1.0	0.3	2.3	Pass
>50 N	Hardness	147	185	147	138	484	171	Pass

**Table 2:** Assay results for the tested brands compared with the innovator product.

	**Samples**					
	**Diodol**	**Enamol**	**Sheladol**	**Cetamol**	**Elymol**	**Panadol (ref)**
Assay BP (90%–110%)	93.6	92.4	91.5	96.6	91.9	93.4
Remarks	Pass	Pass	Pass	Pass	Pass	Pass

**Table 3:** Statistical test for assay results.

**Description**	**Mean**	**Std deviation**	**p** ** value**
Panadol (Std)	93.4	1.12	Ref
Diodol	93.6	1.13	0.84
Enamol	92.4	1.14	0.33
Sheladol	91.5	1.15	0.11
Cetamol	96.6	1.12	0.02
Elymol	91.9	1.14	0.18

**Table 4:** Amount of paracetamol active ingredient released in a dissolution medium.

	**Time (min)**	**Samples**					
		**Diodol**	**Enamol**	**Sheladol**	**Cetamol**	**Elymol**	**Panadol (ref)**
Dissolution (BP >80%)	15	67.40	61.40	95.0	94.90	94.90	97.50
	30	97.80	96.70	97.90	98.00	98.60	99.40
	45	98.40	97.80	99.80	98.70	98.70	99.90
	60	99.60	98.50	100	99.90	100	100
	75	100	99.50	100	100	100	100
	90	100	100	100	100	100	100
	120	100	100	100	100	100	100

Remarks		Pass	Pass	Pass	Pass	Pass	Pass

**Table 5:** Statistical test for dissolution results.

**Description**	**Dissolution (30 min)**	**Std deviation**	**p** ** value**
Panadol (Std)	99.90	1.26	
Diodol	97.8	1.22	0.11
Enamol	96.7	1.19	0.03
Sheladol	97.9	1.23	0.12
Cetamol	98.00	1.24	0.13
Elymol	98.60	1.25	0.27

**Table 6:** Difference and similarity factors between paracetamol generics and innovator product.

**SN**	**Paracetamol brand**	**f1 (0–15)**	**f2 (50–100)**
1	Diodol	5	74
2	Enamol	6	70
3	Sheladol	6	70
4	Cetamol	1	98
5	Elymol	1	99

## Data Availability

All data available are included in the manuscript.
